# The Genomic Load of Deleterious Mutations: Relevance to Death in Infancy and Childhood

**DOI:** 10.3389/fimmu.2015.00105

**Published:** 2015-03-16

**Authors:** James Alfred Morris

**Affiliations:** ^1^Department of Pathology, Royal Lancaster Infirmary, Lancaster, UK

**Keywords:** whole genome sequencing, deleterious mutations, sudden unexpected death in infancy, bacterial toxins, proteomics, molecular autopsy

## Abstract

The human diploid genome has approximately 40,000 functioning conserved genes distributed within 6 billion base pairs of DNA. Most individuals carry a few heterozygous deleterious mutations and this leads to an increased risk of recessive disease in the offspring of cousin unions. Rare recessive disease is more common in the children of cousin marriages than in the general population, even though <1% of marriages in the Western World are between first cousins. But more than 90% of the children of cousin marriages do not have recessive disease and are as healthy as the rest of the population. A mathematical model based on these observations generates simultaneous equations linking the mean number of deleterious mutations in the genome of adults (*M*), the mean number of new deleterious mutations arising in gametogenesis and passed to the next generation (*N*) and the number of genes in the human diploid genome (*L*). The best estimates are that *M* is <7 and *N* is approximately 1. The nature of meiosis indicates that deleterious mutations in zygotes will have a Poisson distribution with a mean of *M* + *N*. There must be strong selective pressure against zygotes at the upper end of the Poisson distribution otherwise the value of *M* would rise with each generation. It is suggested that this selection is based on synergistic interaction of heterozygous deleterious mutations acting in large complex highly redundant and robust genetic networks. To maintain the value of *M* in single figures over many thousands of generations means that the zygote loss must be of the order of 30%. Most of this loss will occur soon after conception but some will occur later; during fetal development, in infancy and even in childhood. Selection means genetic death and this is caused by disease to which the deleterious mutations predispose. In view of this genome sequencing should be undertaken in all infant deaths in which the cause of death is not ascertained by standard techniques.

## Introduction

Infant mortality rates have fallen progressively in UK in the last 50 years, from 18 infant deaths per 1000 live births in 1970 to 4/1000 in 2012 (1). The majority of infant deaths occur in the first month of life (70% in England and Wales in 2012). The post-neonatal mortality rate (infant deaths between 1 and 12 months of age) has also fallen from over 5/1000 live births in 1970 to 1.2/1000 in 2012. The majority of infant deaths after the first month of life are due to sudden unexpected death in infancy (SUDI). The frequency of this condition fell markedly between 1988 and 1995 coinciding with a move from prone to supine sleeping. This followed epidemiological studies, which identified the risk of the prone sleeping position in the early months of life (2, 3). It is one of epidemiology’s great triumphs. The number of infant deaths that remain unexplained after a detailed autopsy has, however, leveled off in recent years and there are still approximately 300 such infant deaths (0.5/1000) each year in England and Wales.

Sudden unexpected death in infancy is more common in infants with young single mothers, or mothers with partners who are unemployed (4). The parents commonly smoke and there is an increased incidence of drug abuse. The infants are less likely to have been breast fed and the parents are less likely to follow national guidelines on safe sleeping practice. SUDI is a condition associated with poor social circumstances and if those conditions could be improved the number of cases would undoubtedly fall. Indeed, the number of cases would fall if all that happened was parents stopped smoking. But it is naïve to believe that the condition could be eliminated in this way. Furthermore, emphasis on poor parenting is of no comfort to those mothers whose infants died in spite of exemplary care and there are many such cases.

In my opinion, the infant autopsy in the twenty-first century should be improved. It should include genome sequencing and analysis of body fluids for bacterial secretory products as well as the standard dissection and histological examination of tissue. This is the molecular autopsy to supplement the standard autopsy. The current methods for assessing infection are inadequate and there is plenty of evidence to indicate that analysis of the genome will provide valuable information in many cases (5). Parents have a right to know why their infant died, and pathologists have a duty to do all that they can to answer that question. Lecturing parents on their inadequacy is no substitute for a full scientific assessment of the cause of death.

In this article, I make a case for the use of whole genome or whole exome sequencing in the infant autopsy. Initially, it will be used for cases where death is unascertained after a detailed standard autopsy. It should also be used in cases of childhood death when the cause is unclear. But eventually, it will be used in all infant deaths because the information supplied will be of considerable value in the broader understanding of the function of human genes as well as for genetic counseling. Genome sequencing should be mandatory in cases of infant death attributed to the shaken baby syndrome, because the evidence for trauma is often weak and some of these cases could be due to natural disease (6). Avoiding a single prosecution will save many multiplies of the $2000 estimated cost of whole genome sequencing.

## Neutral Mutations

If two homologous strands of DNA from an autosomal chromosome from the same person or from a different person are compared over 99% of the bases will be exactly the same (7). The same will apply to the X chromosome and to the Y chromosome when they are compared between individuals. However, discrepancies do occur in approximately 1 in 300 bases and these base changes are due to mutations in previous generations. The rate of mutation when DNA is copied in mitosis is of the order of 5 × 10^−10^ bp per mitotic division (8). The number of bases in the diploid genome is 6 × 10^9^; thus, approximately three bases will be miscopied each time a cell divides. There are over 40 divisions between the zygote and the oocyte, and over 60 between the zygote and spermatozoa. Thus, each individual will have over 100 base changes due to mutations occurring in parental gametogenesis. These are private mutations, they are unlikely to be present in any other member of the human race and they are not shared with siblings.

An individual will also inherit over 100 mutations that arose during grand-parental gametogenesis. These are also private mutations, but they will be present in the genome of parents and will be shared with siblings. Another 100 mutations will have arisen in the gametogenesis of great grandparents, and a further 100 from mutagenesis in great great grandparents, and so on back through thousands of generations. Obviously, grandparents produce twice as many mutations as parents because there are four grandparents and only two parents. But only half of the mutations are passed on in each generation so the number of mutations acquired per individual remains the same at over 100 for each generation.

If the current human variation is 1 in 300 bases and 100 new mutations arise per generation then the process has been going on for 100,000 generations or approximately 2.5 million years. Only 1–4% of the human genome is conserved; this is the protein coding and regulatory part of the genome (7). Thus, the majority of base changes are neutral in evolutionary terms and there is no selection against these mutations. But let us now consider the fate of a new neutral mutation entering the genome of an individual. If that individual has two children then there is a 25% chance that the mutation is not present in the genome of either child; a 25% chance that it is present in the genome of both children and a 50% chance that one child has a copy and the other does not. A similar stochastic process occurs in subsequent generations and the result is that most new neutral mutations are lost purely by chance but a few increase in number from generation to generation, again purely by chance. Fisher was able to show that in a stable population the chance of a new neutral mutation surviving for *n* generations is 2/*n*, when *n* is large. But the number of copies, if it does survive, will be close to *n*/2.

Those neutral mutations that survive will gradually expand in the population over many generations and some will eventually be present in over 1% of the human race. These are the common single nucleotide polymorphisms used in genome wide association studies (GWAS). The majority of these have been in the human genome for over 10,000 generations.

Mutations in protein coding genes and regulatory genes can also be neutral. A base change in a protein coding gene that does not alter the amino acid sequence of the protein is termed a synonymous change. These are commonly neutral. A base change that alters the amino acid sequence is likely to be deleterious but can be neutral. In the latter situation, the new protein is different but works as well as the wild type protein. Or it could be that the new protein is advantageous in certain situations and disadvantageous in others; but neutral overall. Equally, regulatory changes can cause faster or slower and stronger or weaker responses. This, once again, might be advantageous in certain situations and disadvantageous in others. These neutral changes will have similar kinetics to the neutral base changes in non-coding regions described above. The main genetic differences between individuals and between races are due to neutral mutations in protein coding and regulatory genes.

There is some evidence that in certain situations the heterozygote can have an advantage over the homozygote for a particular locus (9). This applies in particular to the HLA system of genes. These loci are highly polymorphic and it appears that the heterozygote is better at dealing with infection and avoiding autoimmune disease than the homozygote. Indeed, bacteria and viruses adapt by evolution to their hosts and new neutral mutations can thwart that adaptation, at least for a few generations. Bacteria adhere to surface proteins on cells and the heterozygote will have fewer proteins of any one type than the homozygote; this again could be advantageous. In certain infections, a strong regulatory response is required, while in others a weaker response might be advantageous. The individual with both responses in their repertoire will be at an advantage.

## Deleterious Mutations

A deleterious mutation is one in which the protein product of a gene is not produced, is produced, and does not function, or is produced and interferes with normal function. Equally, mutations in regulatory elements can be deleterious if the regulatory function is impaired. These mutations can arise from single base changes or more extensive insertions, deletions, or frame shifts. The majority of base changes due to mutation in spermatogenesis and oogenesis lead to neutral mutations in the genome and the best available estimates are that the mean number of new deleterious mutations arising in each generation is between 0.5 and 1.5 (10).

Let us define *M* as the mean number of deleterious mutations in the germ line of adults and *N* as the mean number of new deleterious mutations arising in gametogenesis and passed on to the next generation. Thus, the mean number of deleterious mutations in zygotes is *M* + *N*.

If first cousins marry, they have an increased risk of bearing children with recessive disease. This reflects the presence of heterozygous deleterious mutations in their grandparents that have been passed to both cousins and then appear in the homozygous form in their children. This indicates that we all carry a few deleterious mutations and the value of *M* is >1. But over 90% of the children of cousin marriages do not suffer from a recognizable recessive disease and are as healthy as the rest of the population. This indicates that *M* is a small number and is probably <10 (10).

**Figure 1:**
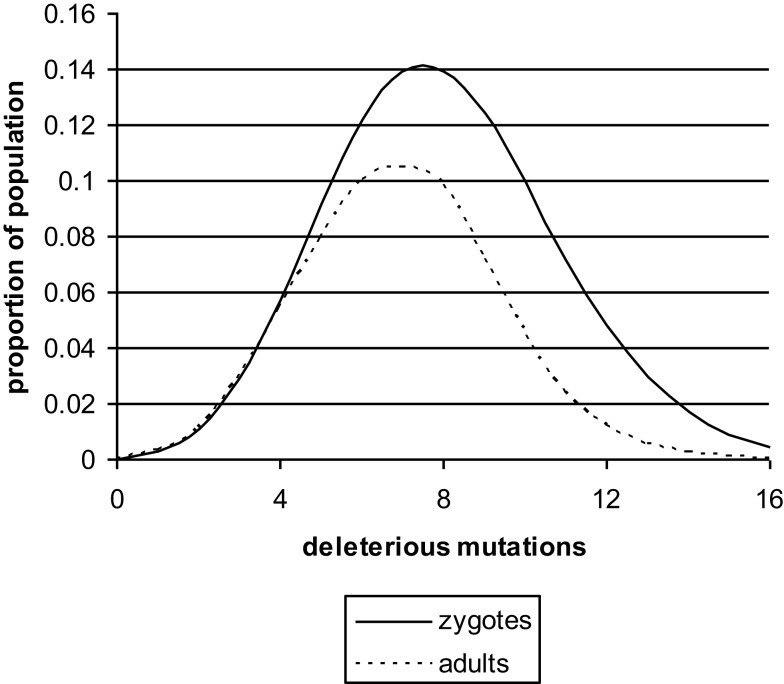
**The distribution of deleterious mutations in zygotes and in adults, based on a previously published theoretical model (13)**. The deleterious mutations interact synergistically to impair the performance of complex genetic systems. Zygote loss will be mainly immediately post conception. The mean number of deleterious mutations (*M*) in UK at present is probably less than shown in the figure. It would fall even further if *N* fell due to improved social conditions.

The nature of meiosis indicates that deleterious mutations will be distributed at random during the formation of spermatozoa and oocytes. Thus, there will be a Poisson distribution of deleterious mutations in zygotes (Figure 1) with a mean <10. A more precise model of this process has been published and the best estimates obtained were *M* = 4–8 and *N* = 0.5–1.5. These estimates were based on the assumption that there are 30,000 genes in the haploid set (10). The current estimate is closer to 20,000 and this means the value of *M* will be proportionally smaller.

If one new deleterious mutation enters the genome per generation and the mean number of deleterious mutations in adults, after many thousands of generations, is still in single figures, then there must be strong selection against the deleterious mutations. This will operate at the upper end of the Poisson distribution. The zygotes that carry the most deleterious mutations will be the least likely to survive and develop into infants and eventually adults. The number of zygotes lost by this process is not inconsiderable. The mean in zygotes is *M* + *N*, and the mean in adults is *M*. To achieve this change, approximately 30% of the zygotes at the upper end of the Poisson distribution will fail to develop (11–13).

The process envisaged, and previously described, is that heterozygous deleterious mutations interact synergistically to impair the performance of large complex genetic systems during development. The individual systems consist of hundreds or thousands of genes. A model along the following lines can explain what is observed (10–13).

- Zygotes, which have four deleterious mutations in an essential genetic system, will not survive. Most of the loss will occur shortly after conception, but a few deaths might arise later in pregnancy or even in early infant life.
- Zygotes, which have three deleterious mutations in an essential system, are likely to survive and be born alive. The system will function adequately most of the time, but will be less robust than normal, and will be at risk of malfunction in response to environmental stress. These infants might die in infancy or childhood due to infection.
- Zygotes with two deleterious mutations in an essential system are likely to survive. Their system will function adequately most of the time and will only be seriously compromised by a major environmental stress. These infants could also die in infancy due to infection.
- Zygotes with one deleterious mutation in an essential system will survive and their system will be robust and will function adequately. They are unlikely to die in infancy.
- Zygotes with zero deleterious mutations have systems, which work beautifully. They grow to be intelligent and healthy adults and have all the luck.

## Gender Differences

Males are more likely to die than females in every year from birth to old age (9, 14). They are also more likely to die *in utero*. In SUDI, there is a constant ratio of three male deaths for every two female deaths (15). The main genetic difference between males and females is that males have only one X chromosome, while females have two. The X chromosome carries approximately 5% of the genome and thus carries in the region of 1000 genes (7). Every male is 1000 genes short of a full set – it explains a great deal.

Female cells, however, express the same number of genes as male cells. This is because one of the X chromosomes in every female cell is inactivated. This is a random process occurring in stem cells. It means that every female is actually two slightly different genetic individuals within one body – this also explains a lot.

Inbreeding depression is an intriguing phenomenon observed in laboratory animals (16, 17). Brother sister mating through many generations leads to genetic homogeneity. Heterogenous changes are gradually lost and each genetic locus contains identical genetic material. These animals, however, are sickly. There is impaired development *in utero*, increased rates of fetal death, low-birth weight, increased infections in early life, and a shortened life-span. There are two possible mechanisms that explain inbreeding depression, one is recessive disease and the other is loss of heterozygous advantage.

1. Recessive disease: there is a selection against deleterious mutations during the process of producing genetic homogeneity and therefore inbred animals have a reduced number of deleterious mutations. However, any deleterious mutations that survive will be homozygous and therefore cause recessive disease.
2. Loss of heterozygous advantage: heterozygous loci confer advantage in fighting infection as discussed above in the section on neutral mutations.

The relevance of inbreeding depression to male death in infancy is that males have the equivalent of inbreeding depression on 5% of the genome. A deleterious mutation on X will cause a recessive disease in males but not in females. In females, only 50% of the cells will express the deleterious gene product. Females will still have heterozygous advantage on X as they are capable of expressing both genes, albeit in different cells.

The number of deleterious mutations on the X chromosome will be approximately *M* ÷ 40, because the X chromosome has 5% of the genome and we are considering the diploid cell. In fact, the number is probably less in males because some deleterious mutations on X will be lethal in the male. The figures in (Table 1) are calculated by assuming that the increased male deaths in SUDI are entirely due to X-linked deleterious mutations. The increased risk of SUDI with a deleterious mutation on X will be between 3- and 11-fold, if the assumption is correct. This indicates the potential value of sequencing the X chromosome in SUDI.

**Table 1:** **This table shows the relative risk of SUDI in males with a deleterious mutation on X, assuming that the excess is caused by sex linked recessive disease**.

Relative risk of SUDI in males with a deleterious mutation on the X chromosome	% of males with a deleterious mutation on the X chromosome	
	SUDI (%)	CONTROLS (%)
3	50	25
6	40	10
11	37	5

*If 25% of control males have a deleterious mutation on X then 50% of SUDI males will carry a deleterious mutation on X and the relative risk of SUDI will rise threefold. Relative risk is the risk of a male with a deleterious mutation on X compared with a male without a deleterious mutation on X. If only 5% of control males have a significant deleterious mutation on X then 37% of SUDI males will have an X-linked mutation and the relative risk will rise 11-fold. The calculations are shown in the Section “Appendix”*.

If X-linked recessive disease is not the explanation, or the sole explanation, then the alternative is loss of heterozygous advantage. This would point to infection as a likely cause and indicates that the molecular autopsy must have two arms: genomics and proteomics.

## Genome Wide Association Studies in Infant Death

In GWAS, up to 500,000 common polymorphisms are assessed in a large cohort of individuals with a specific disease and this is compared with a large cohort of controls from the general population (18–32). Common polymorphisms are those that are present in over 1% of the population. These are neutral changes that have been in the population, without effective selection, for many thousands of generations. Most of the polymorphisms occur in the non-coding regions of the genome but they will be linked to regulatory elements and conserved functioning essential genes. The impetus for these studies was the concept that common mutations cause common disease and the expectation was that there would be strong association between a small number of polymorphisms and the disease under investigation. This has not been borne out in practice. In schizophrenia, manic-depressive psychosis, multiple sclerosis, hypertension, and type 2 diabetes mellitus a large number of polymorphisms are found to be weakly associated with the disease (18–32). The high heritability of schizophrenia and manic-depressive psychosis, for example, is not explained by neutral polymorphisms (28, 29). These results should have been anticipated, because neutral mutations are neutral in evolutionary terms and are not the main genetic cause of disease. Disease is a consequence of deleterious mutations, against which selection operates.

In the GWAS undertaken so far, most of the associations between a polymorphism and a disease have odds ratios <1.5. The significance level used in these studies is set at 0.05 for each polymorphism but since these assays test many hundreds of SNPs there needs to be a correction for multiple testing such that the reporting of false positives (or negatives) is minimized. In view of this large cohorts are required to establish statistical significance and positive associations should be independently checked in a replication set. However, if the odds ratio is raised then this does give a clue to causation. Because it indicates that a linked gene influences the risk of disease. This is particularly relevant if the linked genes have a role in infection or inflammation. Neutral mutations are not the main cause of disease but they can influence the risk of disease by small margins. A neutral mutation can be disadvantageous in relation to one organism but with a compensatory advantage in relation to another; neutral overall in evolutionary terms.

In general, in epidemiological studies, association does not equal causation. But there is an interesting argument that in GWAS association does equal causation. This is because in meiosis there is perfect randomization of neutral polymorphisms and this eliminates confounding factors. The perfect randomized trial. Not everybody agrees with this idea, particularly since it depends on there being no bias in the selection of the control population. However, it does appear that associations, once established, give clues to causation.

I am aware of one GWAS of a cohort of German SIDS cases. This study was funded by the Foundation for the Study of Infant Death (FSID), which has now been renamed the Lullaby Trust. The results have been presented at a number of international meetings but not yet published in full. This study found a number of potential associations with odds ratios between 1 and 1.5. One of the associations reached statistical significance; the odds ratio was 1.5 and the upper bound of the 95% confidence interval was <2. The research workers concerned are planning to enlarge the cohort prior to publication. There are no details at present about the specific loci (33).

Genome wide association studies can also provide information on copy number variants in the experimental and control populations. The larger copy number variants are more likely to indicate deleterious mutations and this can provide useful additional information (34).

There have been a number of studies of cytokine regulatory genes in SIDS (35–40), which are reviewed in detail in an accompanying article (see Ferrante and Opdal) These studies are necessarily smaller than GWAS and do not have the large control cohorts for comparison. There is, however, some evidence that the balance of pro- and anti-inflammatory cytokine responses can influence the response to infection in general and influence the risk of SIDS. Interaction between smoking and the cytokine response is a particular interesting area of investigation. Time will tell whether or not GWAS confirms that cytokine regulatory genes have a role in SIDS.

## Genome Sequencing in Infant Death

It is now possible to sequence the genome at a cost, which is comparable to that of the standard infant autopsy. Whole genome screening or whole exome screening can be undertaken for around $2000 and the price is still falling. This is the investigation, which will reveal the extent to which genetic factors predispose to infant death. The interpretation of the findings, however, will still be extremely difficult, at least initially. Recognizing the small number of deleterious mutations in highly conserved essential genes and distinguishing these from the vast number of other changes in the genome will not be easy, but it is a tractable problem.

In SUDI, the following findings are anticipated:

1. In some cases, a single deleterious mutation in an essential gene will be, in itself, a sufficient explanation for death. We already know that mutations in cardiac channelopathy genes are responsible for approximately 10% of SUDI cases (41). This information has come from sequencing only seven genes. If the entire genome is sequenced it is inevitable that further examples will be found. Some of the mutations will arise *de novo* and some will be present in the germ line of parents. Even in these cases, synergistic interaction with other deleterious mutations might be important and there will be some environmental trigger, such as infection. Recognizing these single deleterious mutations and working out, which are *de novo* will be important for genetic counseling of the families.
2. Single deleterious mutations on X might be a major risk factor for SUDI in males. The male excess in SUDI could be due to sex linked recessives or to a loss of heterozygous advantage as argued above. Sequencing the X chromosome in males is likely to yield a considerable amount of information relevant to infant death.
3. Discerning the role of synergistic interaction of heterozygous deleterious mutations in complex genetic systems will be much more problematical. But it should be possible to determine the actual genetic load of deleterious mutations and relate this to risk of death. Infants with the most deleterious mutations will be at increased risk of death of all types, including SUDI.

## Population Monitoring

The genomic load of deleterious mutations is a major factor in health and disease. These mutations contribute to death in infancy and in childhood. This is an inevitable conclusion of the observation that there is strong selection against these mutations. Selection means genetic death caused by disease to which the mutations predispose.

The rate at which new deleterious mutations enter the genome is a random variable, and many factors will influence the rate. Parental age, smoking, diet, and infection are likely to be involved as is environmental pollution. The models used in this paper indicate that if *N* falls then *M* will also fall over several generations and the population will become healthier. The rate of infant death will fall. There is a public health imperative to measure and monitor the rate at which new deleterious mutations enter the genome so as to recognize causative factors and avoid them as far as possible.

The considerable fall in the rate of infant mortality and overall improvement in health over the last half century will be due to many factors; but it is highly likely that part of this change is a consequence of a fall in rates of somatic mutation in stem cells, including germ cells. Improved social and economic conditions could bring about this change in a number of ways including better diet, less pollution, and less infection. We cannot, however, assume that this process will continue its beneficial course. Economic progress can lead to more pollution not less, and climate change could have many detrimental effects.

## Discussion

In probing the pathogenesis of disease, I follow a simple but powerful maxim: germs cause disease, genes act in complex networks to prevent disease. In so far, as this idea is correct, and it will only be correct in part, we can anticipate that common disease will be due to common organisms. Our attention should therefore focus on bacteria of the normal microbial flora and how they could interact with a genome impaired by deleterious mutations (42, 43).

Whole genome sequencing and whole exome sequencing are new techniques, which were not available in the twentieth century. These techniques should allow us to define precisely the contribution of genetic mutation to infant death. But interpretation of the results of the analysis will not be easy and it will be some time before we are confident in recognizing the significant changes. The cost of genome sequencing is not inconsiderable, but it is comparable with the total current cost of an autopsy. In cases were the cause of infant death is a matter of legal dispute and criminal charges are considered genome sequencing could lead to considerable cost saving (6).

Disease is an interaction between environmental stress and impaired genetic systems. Thus, genome sequencing will not provide a complete answer. It should be supplemented with the other arm of the molecular autopsy, which is proteomic analysis of body fluids. In particular, we need to seek and identify bacterial secretory products in body fluids in order to diagnose infection (5). This article, however, concentrates on the genetic aspects because the genetic techniques are now ready for direct application; the proteomic techniques are still in the phase of development.

A common counter-argument to the ideas presented in this article is that nothing can be done about our genetic constitution and sequencing the genome will not lead to any preventive strategies. In my view, this is a misconception. Deleterious mutations are ultimately caused by environmental factors and if we can identify the factors then the rate of mutation can be decreased. Indeed, falling rates of somatic and germ line mutation are probably a major factor in the improvement in health we have seen over the last 50 years in the technologically advanced countries. Smoking is mutagenic and if we could measure this directly it would be a powerful incentive for young parents, contemplating a family, to quit the habit.

In my experience, the question that parents want answering is “why did my infant die?”; and this takes precedence in their mind over discussions related to preventive strategies. The job of the pathologists is to answer that question. We were unable to do so in many cases in the twentieth century because the techniques of the molecular autopsy were not available. Genome sequencing is now available and should be used. Proteomic techniques will follow in the near future.

## Conflict of Interest Statement

The author declares that the research was conducted in the absence of any commercial or financial relationships that could be construed as a potential conflict of interest.

## Appendix

The calculations are based on the assumption that the excess male death in SUDI is entirely due to heterozygous deleterious mutations on X.

Consider the case in which 25% of males have a deleterious mutation on X.

In 100 cases of SUDI, there will be 60 males and 40 females.

The 20 excess males all have a deleterious mutation on X.

About 25% of the remaining 40 males also have a deleterious mutation on X.

Thus, 30 of 60 males (50%) have a deleterious mutation on X.

About 25% of the male population produces 50% of the deaths.

About 75% of the population produces the other 50% of the deaths.

Relative risk is (50 ÷ 25) ÷ (50 ÷ 75) = 3.

Now consider the case in which 10% of males have a deleterious mutation on X.

In 100 cases of SUDI, there will be 60 males and 40 females.

The 20 excess males all have a deleterious mutation on X.

Just 10% of the remaining 40 males have a deleterious mutation on X.

Thus, 24 of 60 males (40%) have a deleterious mutation on X.

About 10% of the male population produces 40% of the male deaths.

The remaining 90% of the male population produces 60% of the male deaths.

Relative risk is (40 ÷ 10) ÷ (60 ÷ 90) = 6.

Finally, consider the case in which 5% of the males have a deleterious mutation on X.

Twenty-two males of 60 (37%) SUDI deaths will have a deleterious mutation on X.

Thus, 5% of the population of males produces 37% of the deaths.

The remaining 95% of the population produces 63% of the deaths.

Relative risk is (37 ÷ 5) ÷ (63 ÷ 95) = 11.

Please note that the relative risk for specific deleterious mutations will vary markedly.

The calculations relate to the mean of the distribution. The mean is likely to be a constant regardless of time, population and race; hence the remarkable constancy of Mage’s ratio – three male deaths for every two female deaths (15).
